# The Impact of *MEI1* Alternative Splicing Events on Spermatogenesis in Mongolian Horses

**DOI:** 10.3390/ani15233435

**Published:** 2025-11-28

**Authors:** Dailing Song, Guoqing Wang, Terigele Baterin, Yajuan Weng, Manglai Dugarjaviin, Bei Li

**Affiliations:** 1 Key Laboratory of Equus Germplasm Innovation (Coconstruction by Ministry and Province), Ministry of Agriculture and Rural Affairs, Hohhot 010018, China; sdailing@163.com (D.S.); gq101220jy@163.com (G.W.);; 2 Inner Mongolia Key Laboratory of Equus Science Research and Technology Innovation, Inner Mongolia Agricultural University, Hohhot 010018, China; 3 Equus Research Center, College of Animal Science, Inner Mongolia Agricultural University, Hohhot 010018, China; 4 Baotou Municipal Animal Husbandry and Aquatic Products Extension Service Center, Hohhot 014010, China

**Keywords:** *MEI1* gene, alternative splicing, skipped exons, mutually exclusive exons, spermatogenesis

## Abstract

Background: Normal spermatogenesis in Mongolian horses depends on the mitotic division of spermatogonia, two successive meiotic divisions, and the morphological transformation of spermatids into mature spermatozoa. The *MEI1* gene is involved in the meiosis cycle and is required for normal chromosome association during meiosis. Previous studies have shown that alternative splicing of *MEI1* may promote spermatogenesis in Mongolian horses. In this paper, the regulatory effects of different *MEI1* alternative splicing events on Mongolian horse spermatogenesis are investigated. Results: In this study, two overexpressed lentiviral vectors with mutually exclusive exon (MXE) and skipped exon (SE) events of *MEI1* were constructed and successfully used to infect Sertoli cells. After 72 h of viral infection, the expression of *MEI1* was higher in the SE event than in the MXE event (*p* < 0.001), as shown by fluorescence quantification; transcriptomics and metabolomics were then used to screen and annotate the differential genes and metabolites, and 193 differentially expressed genes (comprising 109 genes, such as *MEI1*, and 84 genes with upregulated and downregulated expression, respectively) and 11,360 differentially expressed metabolites (comprising 7494 and 3866 metabolites with upregulated and downregulated expression, respectively) were screened. Differential genes and metabolites were mainly enriched in several metabolic pathways related to spermatogenesis. Differential genes such as *IL31RA*, *ATP2B3*, and *CASQ2* were highly expressed in SE events, while *IL11*, *PRLR*, and *CCR7* were highly expressed in MXE events. Metabolites such as folic acid and spermine were highly expressed during SE events, while citric acid and glutathione were highly expressed during MXE events. This suggests that both MXE and SE events of the *MEI1* gene can promote the activity of the spermatogenesis signaling pathway. Conclusions: The MXE and SE splicing events of the *MEI1* gene may influence spermatogenesis by regulating the expression of spermatogenesis-related genes and metabolites. These findings provide a theoretical foundation for further investigations into the regulatory mechanisms of different alternative splicing events in Mongolian horse spermatogenesis.

## Simple Summary

This study examines how two forms of the *MEI1* gene—exon skipping (SE, omitting a gene segment) and mutually exclusive exons (MXE, selecting one of two exons)—affect sperm production in Mongolian horses. Using viral carriers, we introduced these splicing types into Sertoli cells (SCs) and found that SE-type *MEI1* showed higher expression than MXE-type (*p* < 0.001). Transcriptomic and metabolomic analyses identified 193 differentially expressed genes (109 upregulated, 84 downregulated) and 11,360 differential metabolites (7494 increased, 3866 decreased). These were mainly enriched in sperm formation pathways: SE elevated genes (*IL31RA*, *ATP2B3*, *CASQ2*) and metabolites (folic acid, spermine), while MXE increased genes (*IL11*, *PRLR*, *CCR7*) and metabolites (citric acid, glutathione). Both splicing forms activated sperm production pathways by regulating specific genes and metabolites, revealing a new role for *MEI1* in equine reproduction and providing a basis for improving reproductive efficiency and genetic conservation in Mongolian horses.

## 1. Introduction

The testis is the primary reproductive and endocrine organ in male mammals, responsible for sperm production and androgen secretion [1]. Its seminiferous tubules, composed of germ cells, Sertoli cells, and peritubular myoid cells, are the site of spermatogenesis [2]. Sertoli cells play a central role by providing structural support, nutritional factors, and androgen-binding proteins to maintain high testosterone levels and promote germ cell maturation [3,4]. They also form the blood-testis barrier, safeguarding the microenvironment required for germ cell development [5,6]. Spermatogenesis proceeds through spermatogonial mitosis, spermatocyte meiosis, and spermatid metamorphosis, culminating in the production of mature sperm. Meiosis, involving DNA replication followed by two successive divisions, ensures chromosome number stability and is essential for normal sperm formation [7,8].

The meiotic double-strand break forming protein 1 (*MEI1*) gene, initially identified through chemical mutagenesis of embryonic stem cells in infertile mouse models, is located on chromosome 22 in humans and chromosome 15 in mice. It is essential for normal meiotic chromosome synapsis in vertebrates [9,10]. During meiosis, *MEI1* promotes the formation of DNA double-strand breaks (DSBs), while the germ cell-specific zinc finger protein ZFP541 suppresses DSB generation. The balanced interplay between *MEI1* and *ZFP541* ensures the proper progression of germ cell development [11]. Studies using *MEI1*-deficient models reveal its critical role in gametogenesis. Nguyen et al. observed that most *MEI1*-null oocytes displayed aberrant spindle morphology and chromosome misalignment. Approximately 63% of these oocytes failed to extrude the first polar body, while an additional 20% released morphologically abnormal polar bodies. In some cases, all chromosomes and spindle microtubules were extruded into a polar body, resulting in an anucleate oocyte [12]. In males, *MEI1* expression is testis-specific. Sato et al. identified mutations in the *MEI1* coding region that cause complete meiotic arrest at early stages, leading to azoospermia—highlighting the gene’s indispensable role in spermatogenesis [13]. More recently, Liu et al. reported a novel homozygous exon 19 deletion in *MEI1* within a consanguineous family, which caused azoospermia in one affected male and early embryonic arrest in a female carrier. Functional analyses revealed that this deletion leads to exon skipping, produces a truncated protein, disrupts meiotic chromosome synapsis, and generates aneuploid gametes with severely impaired developmental potential beyond the blastocyst stage [14]. Emerging evidence indicates that germ cells can influence Sertoli cell function [15]. Given the pivotal role of *MEI1* in meiotic regulation, we hypothesize that this gene may indirectly contribute to the functional modulation of Sertoli cells through its effects on germ cells. Cui et al. [16]. found that the *MEI1* isoform resulting from a splicing event (SE) influences the proliferation of Sertoli cells, suggesting a potential role for *MEI1* in the bidirectional communication between germ cells and Sertoli cells. Nevertheless, the precise molecular mechanisms underlying this regulatory pathway remain to be fully elucidated. Moreover, although *MEI1* is well-established as a germ cell–specific factor, its expression in Sertoli cells and the functional relevance of its splicing isoforms in these cells remain largely unexplored.

Alternative splicing is a fundamental post-transcriptional mechanism that greatly expands proteomic diversity. During this process, the spliceosome recognizes different splice sites on precursor mRNA, leading to the excision of introns and the variable joining of exons. This results in multiple distinct mRNA transcripts from a single gene, serving as a crucial regulatory layer for gene expression in eukaryotes [17,18]. It is estimated that alternative splicing occurs in approximately 95% of human genes and 65% of multi-exon mouse genes [17,18]. Common types of alternative splicing events include skipped exons (SE), mutually exclusive exons (MXE), alternative 5′ and 3′ splice sites, and retained introns (RI) [19]. Accumulating evidence underscores the pivotal role of alternative splicing in testicular development and spermatogenesis. An analysis of RNA-Seq data from sheep testes across four developmental stages revealed that the vast majority of testis-expressed genes undergo alternative splicing, with a significantly higher frequency of splicing events in sexually mature testes compared to neonatal and pubertal stages, highlighting its importance during testicular maturation [20]. Furthermore, Guan et al. demonstrated that undernutrition-induced defects in spermatogenesis and increased germ cell apoptosis are closely associated with altered mRNA expression and pre-mRNA splicing patterns [21]. The germ cell-specific factor Esrp1 has been shown to regulate spermatogonial differentiation through alternative splicing [22]. Similarly, the steroidogenic acute regulatory protein (StAR) and its splicing variant StAR-a, which are enriched in Leydig cells, are essential for testosterone biosynthesis and subsequent spermatogenesis [23]. Collectively, these studies establish alternative splicing as a critical regulator of normal spermatogenesis in mammals. However, the functional consequences of the vast majority of testis-specific splicing events, particularly those in key meiotic genes like *MEI1*, remain largely unexplored. A systematic understanding of how specific splicing isoforms regulate meiotic progression and germ cell development is still lacking.

The Mongolian horse is one of the most famous local breeds in China and the world. It is characterized by cold resistance, resistance to rough feeding, disease resistance, and strong gregariousness. Liu et al. [24]. compared key developmental indicators of testicular tissue in Mongolian horses before and after sexual maturation. Their results demonstrated that the diameter and cross-sectional area of seminiferous tubules, as well as the number of spermatogenic epithelial cells, were significantly greater in sexually mature horses compared to immature individuals. At the molecular level, the expression levels of spermatogonial marker genes (*ITGA6*, *DAZL*, *FGFR3*) and Sertoli cell marker genes (*GDNF*, *KITLG*) were also significantly upregulated in the testicular tissue of sexually mature Mongolian horses. In mammals, *MEI1* is closely linked to the meiotic process involved in spermatogenesis. In our previous study, we found that the *MEI1* gene in the testicular tissue of sexually mature Mongolian horses has two alternative splicing events: SE and MXE, suggesting that alternative splicing of the *MEI1* gene may be related to spermatogenesis [25]. The occurrence of SE events in *MEI1* is conducive to the regulation of spermatogenesis in Mongolian horses [16]. This study aims to elucidate the indirect role of different MEI1 alternative splicing isoforms in Mongolian horse spermatogenesis via Sertoli cell-focused transcriptomic and metabolomic analyses. The findings are expected to establish a theoretical basis for employing molecular breeding techniques to enhance semen quality and sperm quantity, thereby improving reproductive efficiency in horses.

## 2. Materials and Methods

### 2.1. Test Material

Bilateral testicular samples were collected from three 2-year-old healthy Mongolian horses maintained under identical rearing conditions at a breeding farm in Hohhot, Inner Mongolia Autonomous Region. The isolation and culture of primary Sertoli cells were performed according to the previously established protocol for Mongolian horse testicular tissues [26]. Cell purification was achieved through differential plating, with medium replacement using fresh culture medium at 6 h post-seeding and subsequently every 24 h. When Sertoli cells reached 80% confluence, they were passaged and cultured until the third generation for subsequent experiments.

### 2.2. Construction of Overexpression Lentiviral Vectors

We designated the *MEI1* transcript undergoing the MXE event as MEI1(MXE) and the one undergoing the SE event as MEI1(SE). The subsequent lentiviral vector construction and viral titer determination were completed by GENEWIZ Biotechnology Co., Ltd. (Suzhou, China). Primers were designed based on the Equus caballus *MEI1* CDS sequence from the NCBI database. The target fragments were amplified by PCR, digested with restriction enzymes, and gel-purified. Each fragment was ligated into the corresponding lentiviral vector (pGWLV12-mcherry for MEI1(MXE); pGWLV10-BFP for MEI1(SE)) using T4 DNA ligase. The ligated products were transformed into competent cells, and positive clones were selected and verified by restriction digestion. Subsequently, large-scale plasmid extraction, endotoxin removal, lentiviral packaging, and concentration were performed. The final titers of the recombinant lentiviruses were determined to be 6.69 × 10^8^ TU/mL for MEI1(MXE) in pGWLV12(mcherry) and 1.78 × 10^9^ TU/mL for MEI1(SE) in pGWLV10-BFP, both suitable for subsequent experiments.

Viral titer was determined as follows:

- HT1080 cells were seeded in 24-well plates at 8.0 × 10^4^ cells per well and cultured for 24 h at 37 °C under 5% CO_2_;
- Viral stock was serially diluted to 50 µL, 0.5 µL, and 0.05 µL gradients;
- Culture medium was replaced with MEM containing 4% FBS and the diluted virus; each dilution was assayed in duplicate;
- After 24 h of infection, the medium was replaced with fresh medium;
- Genomic DNA was extracted 72 h post-infection, and viral titer was determined by quantitative PCR.

### 2.3. Lentiviral Infection of Sertoli Cells

Third-generation Sertoli cells were inoculated into six-well plates, and lentiviral infection was performed when the cells were approximately 30% confluent. The individual steps are as follows. First, the old culture solution was aspirated and washed two to three times with double antibody-free DPBS, and 1 mL of DMEM-F12 medium was added to each well. The virus was diluted according to the calculated volume specified in the GENEWIZ Lentiviral Transduction Protocol, after which LV assistant co-transformant was added and mixed by stirring in an “8” shape several times until the virus was well mixed with the medium. After 6 h of incubation, a small amount of medium was added to replenish the liquid in the wells to 2 mL, and the incubation was continued at a constant temperature. After the cells were infected with the virus for 24 h, the liquid in the wells was aspirated and washed two to three times with double antibody-free DPBS and then replenished with a fresh culture medium of normal cultured cells. The infection efficiency was observed by fluorescence microscopy and photographed and recorded after 24, 48, and 72 h of infection.

### 2.4. Detection of MEI1 Expression in the MXE and SE Groups by qRT-PCR at 72 h

Total RNA was extracted from the SE, MXE, and SC groups using the AllPrep^®^ DNA/RNA Mini Kit (QIAGEN, Hilden, Germany). The reverse transcription reaction mixture was prepared according to the manufacturer’s instructions (Table S1). After adding all components into a microcentrifuge tube, the mixture was thoroughly vortexed. The reverse transcription reaction was carried out under the conditions specified in Table S2. The concentration and purity of the synthesized cDNA were measured using a microvolume spectrophotometer. All cDNA products were stored at −20 °C for subsequent use. Gene-specific primers were designed based on the target sequences and synthesized by Shanghai Bioengineering Co., Ltd. (Shanghai, China) (Table S3). The primer powders were centrifuged and reconstituted with ddH_2_O to a final concentration of 10 μmol/μL as stock solutions. The qPCR reaction mixture was prepared following the instructions of the SYBR Green qPCR kit, with *GAPDH* used as the internal reference gene. Each sample was run in triplicate, and amplification was performed on a real-time PCR instrument according to the established protocol (Tables S4 and S5).

### 2.5. Transcriptome Analysis and Data Processing

The SE and MXE groups each included three biological replicates, with approximately 1 × 10^7^ cells per replicate. Total RNA was extracted from the samples using TRIzol reagent according to the manufacturer’s protocol. Comprehensive quality control was performed to ensure RNA integrity and purity: RNA concentration and purity were assessed using a NanoDrop ND-1000 spectrophotometer (NanoDrop, Wilmington, DE, USA), while RNA integrity was evaluated with a Bioanalyzer 2100 (Agilent, Santa Clara, CA, USA). All samples met the predefined quality thresholds (Table S6) before proceeding to library construction. PolyA-containing mRNAs were enriched using oligo (dT) magnetic beads and fragmented using a magnesium ion interruption kit. The fragmented RNA was then transcribed into cDNA through reverse transcription by *E. coli* DNA polymerase I (NEB, cat.m0209, Ipswich, MA, USA) and RNase H (NEB, cat.m0297, Ipswich, MA, USA). dUTP Solution (Thermo Fisher, cat.R0133, Waltham, MA, USA) was used to complete the ends of the double-stranded DNA and add an A base at each end. The fragments were size-selected and purified using magnetic beads. The second strand was digested with the enzyme UDG (NEB, cat.m0280, Ipswich, MA, USA) and amplified by PCR to obtain fragments of 300 bp ± 50 bp. The PCR conditions were as follows: pre-denaturation at 95 °C for 3 min, 8 cycles of denaturation at 98 °C for 15 s, annealing at 60 °C for 15 s, extension at 72 °C for 30 s, and final extension at 72 °C for 5 min. Library quality was verified using the Bioanalyzer 2100 system, and only libraries passing quality control were used for subsequent sequencing. Paired-end sequencing was performed on an Illumina NovaseqTM 6000 platform (LC Bio Technology Co., Ltd., Hangzhou, China). The Cutadapt software (v5.0) was used to filter the raw sequencing data and obtain clean reads (Table S7). HISAT2 (v2.2.1) was employed to align the clean reads to the reference genome (Table S8). StringTie (v2.1.3) was used to assemble the mapped reads for each sample, and Gffcompare (v0.12.6) was utilized to merge and reconstruct the transcriptomes of all samples. The Ballgown package (v2.26.0) was applied to estimate expression levels and calculate FPKM values. DESeq2 (v1.42.0) was used to identify differentially expressed genes between the two groups, with the criteria set as *p*-value < 0.05 and |FC| ≥ 2. Finally, GO functional and KEGG pathway enrichment analyses were performed on the differentially expressed genes. Pathway information was obtained from the KEGG database (https://www.kegg.jp/). Usage was approved by KEGG via email on 15 May 2025.

### 2.6. Metabolomics Analysis and Data Processing

SE and MEX groups each contained six replicates, with approximately 1 × 10^7^ cells per replicate, and metabolites were extracted from the samples via protein precipitation using an organic reagent. The metabolite samples were stored in a refrigerator at −80 °C before sampling. Samples were collected using an LC-MS system, according to the manufacturer’s instructions. Chromatograms were separated using a Thermo Scientific UltiMate 3000 HPLC (Thermo Fisher Scientific, Waltham, MA, USA) and an ACQUITY UPLC BEH C18 (Waters Corporation, Milford, MA, USA) column in the reverse phase. During collection, the column temperature was maintained at 35 °C, and the flow rate was 0.4 mL/min. The mobile phases used were as follows: Phase A, water (0.1% formic acid); Phase B, acetonitrile (0.1% formic acid). The gradient elution conditions were: 0–0.5 min, 5% B; 0.5–7 min, 5–100% B; 7–8 min, 100% B; 8–8.1 min, 100–5% B; 8.1–10 min, 5% B. Metabolites eluted from the column were detected using a Q-Exactive high-resolution tandem mass spectrometer (Thermo Fisher Scientific, Bremen, Germany). Precursor spectra (70–1050 *m*/*z*) were collected at a resolution of 70,000 to achieve an AGC target of 3e6 with a maximum injection time of 100 ms. Fragmentation spectra were collected at a resolution of 17,500 to achieve an AGC target of 1 × 10^5^, with a maximum injection time of 80 ms. Quality control samples were collected after every 10 samples to evaluate the stability of LC-MS.

Proteowizard MSConvert software (v3.0.20286) was used to convert downstream raw data into readable data. The XCMS software (v3.16.0) was used for peak extraction and quality control (Figure S1). CAMERA (v1.52.0) was used for the addition and ion annotation of the extracted substances. Metabolite identification, quantification, and differential metabolite screening were performed using metaX software (v1.6.1). Metabolites were annotated using the HMDB 5.0, KEGG, and other databases. Univariate analysis of the fold-change and *t*-test statistical tests were used, and the BH correction was used to obtain q-values. Based on the VIP values obtained by multivariate statistical analysis using PLS-DA, differentially expressed metabolites were screened.

### 2.7. Combined Transcriptome and Metabolome Analysis

An integrated analysis of transcriptomic and metabolomic data was performed to elucidate the interactions between differential genes and metabolites. First, Spearman correlation coefficients between differentially expressed genes and differentially accumulated metabolites were calculated using the cor program in R (v4.5.2). To visually represent the association patterns, a nine-quadrant plot (Figure S2) was generated to display the fold changes of genes and metabolites with a strong correlation (Spearman coefficient > 0.8) across comparison groups. Subsequently, pathway enrichment analysis was conducted using the KEGG database to identify biological pathways commonly shared by the differential genes and metabolites within the same group. Finally, MetScape software (v3.1.3) was utilized to construct and visualize the interaction networks between key genes and metabolites within the significantly enriched metabolic pathways.

### 2.8. Subcellular Localization of MEI1 in Sertoli Cells

Immunofluorescence staining was performed to determine the expression and localization of MEI1 protein in primary Mongolian horse Sertoli cells. The procedure was as follows:

(1) Cell seeding: Third-passage Sertoli cells were seeded on coverslips in 24-well plates. After 24 h of culture, when cells reached approximately 50% confluence, they were washed three times with PBS (5 min each) on a shaking platform.
(2) Fixation: Cells were fixed with 4% paraformaldehyde at room temperature for 30 min, followed by three PBS washes (5 min each).
(3) Permeabilization: Cells were permeabilized with 0.1% Triton™ X-100 at room temperature for 30 min, then washed three times with PBS.
(4) Blocking: Blocking was carried out with 5% BSA in PBS at room temperature for 1 h.
(5) Primary antibody incubation: After removal of blocking solution, cells were incubated overnight at 4 °C with anti-MEI1 mouse monoclonal antibody (1:200) diluted in 5% BSA.
(6) Secondary antibody incubation: The primary antibody was recovered, and cells were washed three times with PBS, followed by incubation with Alexa Fluor™ 488-conjugated goat anti-mouse IgG (1:1000) at room temperature for 1 h in the dark. Cells were then washed three times with PBS.
(7) Nuclear staining: Cells were stained with DAPI (1:500 in PBS) for 10 min in the dark and washed three times with PBS.
(8) Mounting: Coverslips were mounted onto glass slides using an anti-fade mounting medium, avoiding air bubbles, and sealed with nail polish.
(9) Imaging: Images were acquired using a laser scanning confocal microscope.

### 2.9. Prediction of Higher-Order Structures of MEI1 Splice Isoforms

(1) Nucleotide sequences of the two *MEI1* splice variants were translated into amino acid sequences using the ExPASy Translate tool (https://web.expasy.org/translate/, accessed on 24 November 2025);
(2) Secondary structures were predicted using SIMPA96 (https://npsa.lyon.inserm.fr/cgi-bin/npsa_automat.pl?page=/NPSA/npsa_simpa96.html, accessed on 24 November 2025);
(3) Tertiary structures were modeled via the Phyre2 online server (http://www.sbg.bio.ic.ac.uk/phyre2, accessed on 24 November 2025).

## 3. Results

### 3.1. High-Order Structural Predictions for MEI1 Alternative Splicing Isoforms

#### 3.1.1. Secondary Structure Prediction

**Figure 1:**
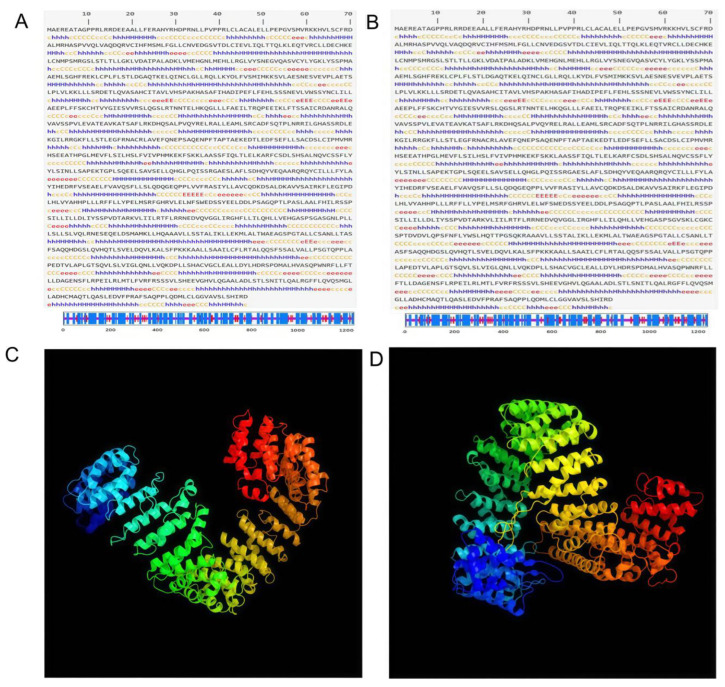
High-Order Structural Predictions of MXE and SE Protein Isoforms. (**A**,**B**) Secondary structure predictions of the MXE and SE isoforms. The predicted secondary structural elements are color-coded as follows: blue α-helices (H), red for β-strands (E), and yellow/orange for random coils (**C**,**D**) Predicted tertiary structures of the SE and MXE proteins generated by Phyre2.The rainbow coloring from blue to red denotes the orientation from the N-terminus to the C-terminus.

The nucleotide sequences corresponding to the two *MEI1* splice variants were first translated into amino acid sequences using the ExPASy Translate tool. These protein sequences were then analyzed with the SIMPA96 module to predict their secondary structures. In the MXE isoform (Figure 1A), α-helices constitute 57.15% of the structure, extended strands (β-sheets) account for 9.54%, and random coils make up 33.23%. In contrast, the SE isoform contains 55.13% α-helices, 10.01% β-sheets, and 34.79% random coils (Figure 1B). Thus, α-helical and random-coil elements predominate in both *MEI1* variants.

#### 3.1.2. Tertiary Structure Prediction

We next used the Phyre2 server to model the three-dimensional structures of each isoform. Comparison of the resulting models reveals that alternative splicing–induced sequence changes indeed alter the spatial conformation of *MEI1*, demonstrating that SE and MXE events can modulate the protein’s tertiary fold (Figure 1C,D).

### 3.2. Dual Verification of MEI1 Nuclear Localization and Expression in Sertoli Cells

#### 3.2.1. Construction of Two Overexpression Lentiviral Vectors, MEI1 (MXE) in pGWLV12 (Mcherry) and MEI1 (SE) in pGWLV10-BFP

The synthesized *MEI1* gene was inserted via the cloning sites *BamH I* (GGATCC) and *Nsi*-*I* (ATGCAT) into the empty vector pGWLV12 (mcherry) with a red fluorescent marker. The synthesized *MEI1* gene was incorporated into the empty vector pGWLV10-BFP with a blue fluorescent marker through the cloning sites *Nhe I* (GCTAGC) and *Nsi*-*I* (ATGCAT). Two overexpressed lentiviral vectors with MXE and SE events of *MEI1* were constructed. The results of the two vectors were detected by agarose gel electrophoresis. The length of the target bands matched the length of the target gene fragments, showing that *MEI1* (MXE) in pGWLV12 (mcherry) and *MEI1* (SE) in pGWLV10-BFP vectors were successfully constructed. Subsequently, maps of the two overexpressed lentiviral vectors MEI1 (MXE) in pGWLV12 (mcherry) and MEI1 (SE) in pGWLV10-BFP were created (Figures S3 and S4). The total lengths of the vectors MEI1 (MXE) in pGWLV12 (mcherry) and MEI1 (SE) in pGWLV10-BFP were 12,245 bp and 12,278 bp, respectively, with the lengths of *MEI1* being 3745 bp and 3717 bp, respectively.

#### 3.2.2. Two Overexpression Lentiviral Vectors for Infection of Mongolian Equine Testicular Sertoli Cells

**Figure 2:**
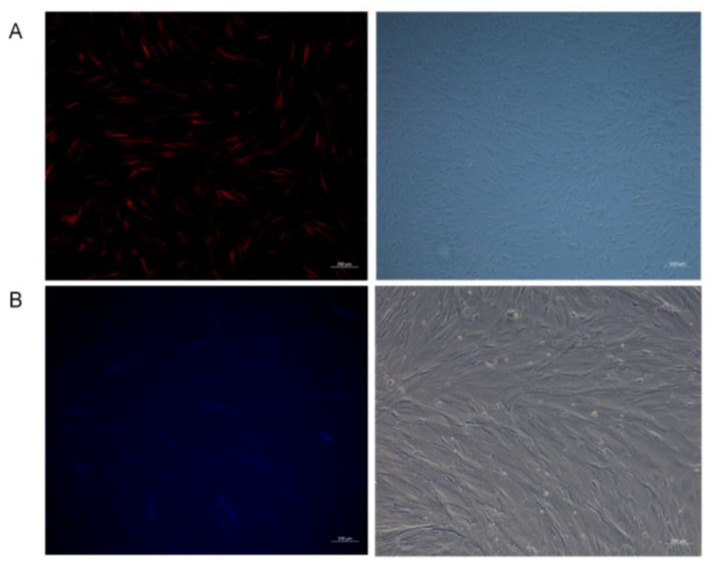
Virus infects the cells for 72 h. (**A**,**B**) Phase contrast and corresponding green fluorescent images of cells at 72 h post-transfection with the *MEI1* (MXE) and (SE) isoform vectors, respectively.

MEI1 (MXE) in pGWLV12 (mcherry) and MEI1 (SE) in pGWLV10-BFP lentiviral vectors were used to infect Sertoli cells for 24 h and then replaced with a complete culture medium in a constant temperature incubator. Infection efficiency was observed by inverted fluorescence microscopy after 24, 48, and 72 h of viral infection. The results were recorded photographically. The results showed that the efficiency of viral infection was the highest after 72 h, being at approximately 80% for MEI1 (MXE) in pGWLV12(mcherry) and 60% MEI1 (SE) in pGWLV10-BFP (Figure 2A,B).

#### 3.2.3. Localization of the MEI1 Gene in Sertoli Cells

**Figure 3:**
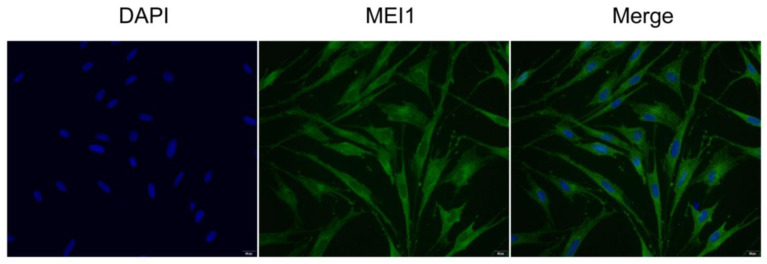
*MEI1* gene expression in sertoli cells. Immunofluorescence staining showing the localization of MEI1 protein (green) in third-generation Mongolian horse Sertoli cells. Nuclei were counterstained with DAPI (blue). The merged image demonstrates the co-localization of MEI1 within specific subcellular compartments.

The expression and subcellular localization of MEI1 protein in Sertoli cells were detected using cellular immunofluorescence (Figure 3). The results demonstrated clear nuclear localization of MEI1 protein in Sertoli cells, providing cytological evidence for the role of MEI1 in this cell type and supporting the use of Sertoli cells for functional verification experiments of the *MEI1* gene.

#### 3.2.4. Detection of MEI1 Expression in the MXE and SE Groups by qRT-PCR

**Figure 4:**
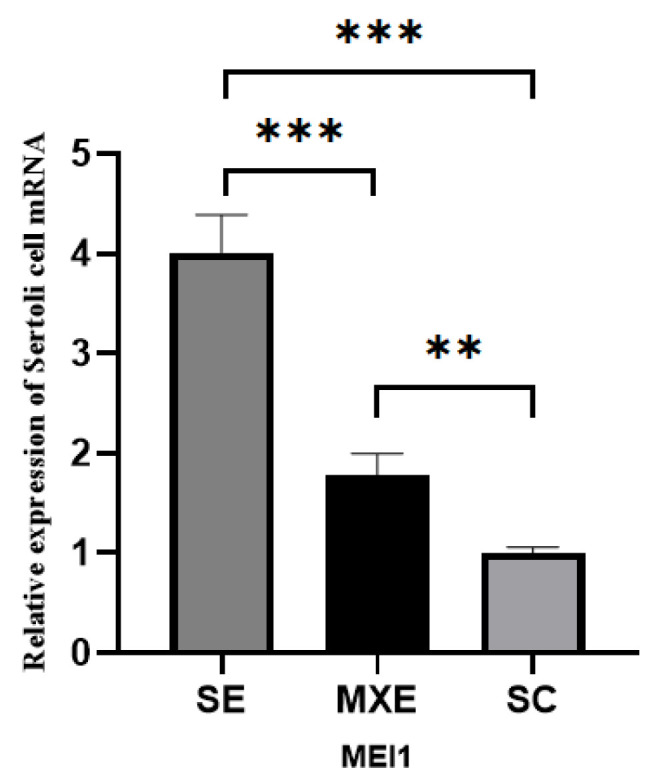
Detection of *MEI1* expression in the MXE and SE groups by qRT-PCR. *MEI1* expression is significantly altered upon alternative splicing perturbation. Relative mRNA expression levels of *MEI1* were quantified by RT-qPCR in three cell groups: Control (normal cells), cells expressing the SE isoform, and cells expressing the Mxe isoform. Data are presented as mean ± SEM from three independent experiments. **, *p* < 0.01; ***, *p* < 0.001 versus the Control group.

The qRT-PCR analysis of *MEI1* gene expression across the SC, MXE, and SE groups demonstrated that both the MXE and SE groups exhibited significantly higher expression levels compared to the SC group (*p* < 0.01 and *p* < 0.001, respectively). Furthermore, the SE group showed a significant increase in *MEI1* expression relative to the MXE group (*p* < 0.001) (Figure 4).

### 3.3. Divergent Roles of MEI1 SE and MXE Isoforms Revealed by Comparative Transcriptomics in Sertoli Cells

#### 3.3.1. Results of Differential Expression Gene Screening

**Figure 5:**
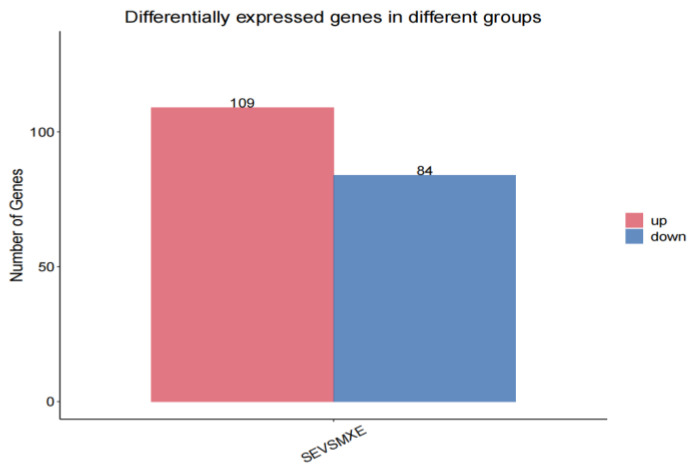
Differential gene frequency histogram. The bar graph quantifies the total number of upregulated (red) and downregulated (blue) genes identified through RNA-seq analysis. The numerical values on the bars represent the exact count of DEGs.

Sertoli cells infected with MEI1 (MXE) in pGWLV12 (mcherry), called MEI1 (MXE), were used as the control group. Sertoli cells infected with MEI1 (SE) in pGWLV10-BFP (referred to as MEI1 (SE)) were used as the test group. Differentially expressed genes were screened using the DESeq2 software (V1.50.0) with |log2fc| ≥ 1 and q < 0.05. A total of 193 differentially expressed genes were identified, of which 109 had their expression levels upregulated, including the *MEI1* gene, and 84 had their expression levels downregulated (Figure 5).

#### 3.3.2. Cluster Analysis of Differentially Expressed Genes

**Figure 6:**
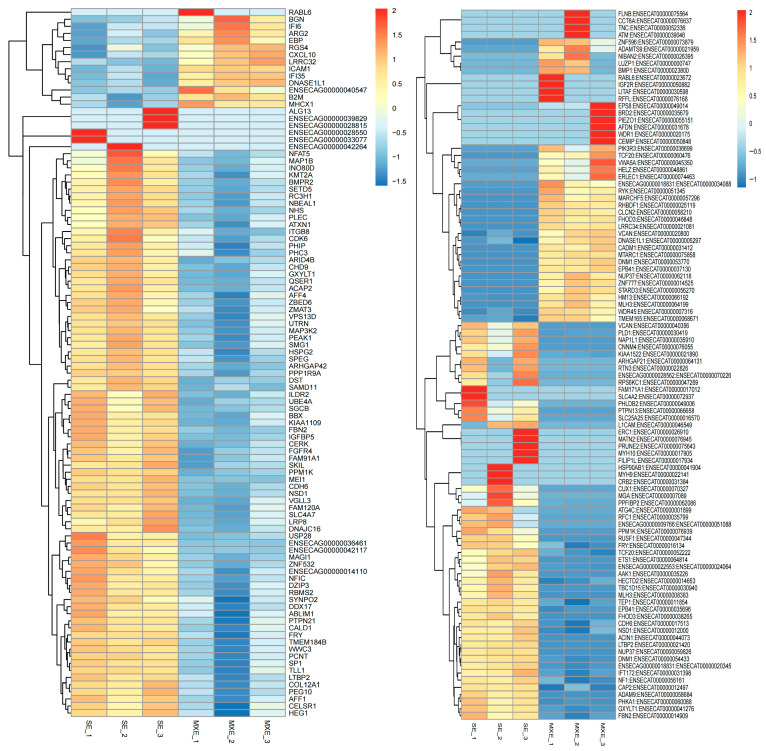
Cluster Analysis of Differentially Expressed Genes. The top 100 genes and corresponding transcripts with the smallest *p*-values were selected for hierarchical clustering analysis. Expression patterns are visualized across samples infected with two different *MEI1*-overexpressing lentiviruses (MXE and SE variants), with normalized expression levels represented by color intensity.

Hierarchical clustering of differentially expressed genes was performed based on the similarity of gene expression profiles across samples, providing a visual representation of expression patterns. A heatmap (Figure 6) was constructed using the top 100 genes and their corresponding transcripts with the smallest *p*-values, with samples plotted along the horizontal axis and genes along the vertical axis. This approach allowed for a comprehensive analysis of global gene expression changes in cells infected with the two *MEI1*-overexpressing lentiviruses.

#### 3.3.3. GO Enrichment Analysis of Differentially Expressed Genes

**Figure 7:**
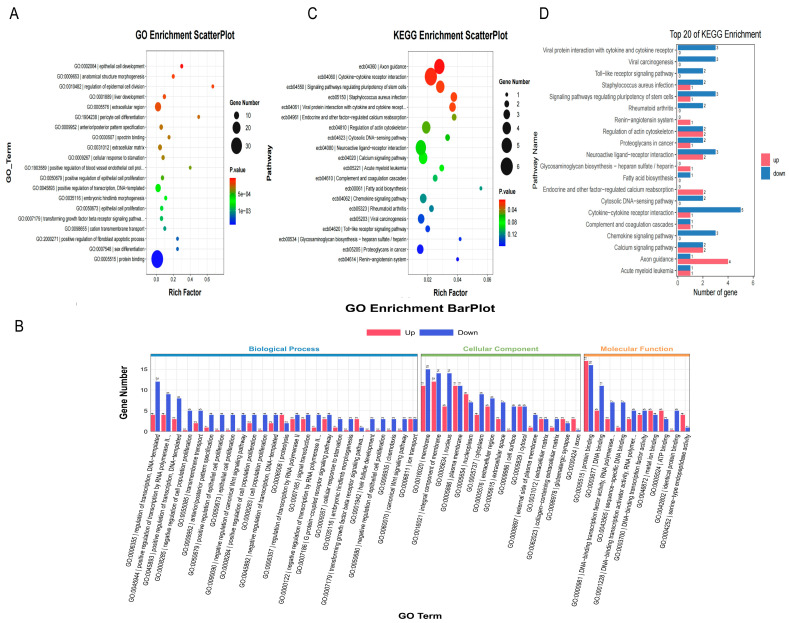
GO and KEGG enrichment analyses of differentially expressed genes. (**A**) Scatter plot of the top 20 GO terms, where dot size and color represent gene count and enrichment significance, respectively. (**B**) Bar plot of the top 20 GO terms, categorized by ontology (BP, CC, MF) and gene regulation direction. (**C**) Scatter plot of the top 20 KEGG pathways, with dot size and color indicating gene count and enrichment significance. (**D**) Bar plot of the top 20 KEGG pathways, showing enrichment significance and the number of DEGs for each pathway. Due to space constraints leading to label truncation in the figure, the full names of the key enriched terms are listed below:GO:1903589: positive regulation of blood vessel endothelial cell proliferation; GO:0007179: transforming growth factor beta receptor signaling pathway; GO:0045944: positive regulation of transcription by RNA polymerase II; GO:0000122: negative regulation of transcription by RNA polymerase II; GO:0000981: DNA-binding transcription factor activity, RNA polymerase II-specific; GO:0001228: DNA-binding transcription activator activity, RNA polymerase II-specific; ecb04061: Viral protein interaction with cytokine and cytokine receptor.

Gene Ontology is an internationally standardized classification system for gene functions whose basic unit is the term. GO has three ontologies: molecular function, cellular component, and biological process. GO enrichment analysis was performed to investigate the function of the different genes in the testis. It was found that the term with significant enrichment of differential genes had entries related to the process of spermatogenesis, such as epithelial cell proliferation, epithelial cell development, regulation of epithelial cell division, positive regulation of transcription, and protein binding (Figure 7A). Subsequently, the enrichment of upregulated and downregulated differential genes in the three GO ontologies was further analyzed (Figure 7B). It was found that differential genes were mainly enriched in “cellular component,” followed by “molecular function” and finally “biological process.” Differentially expressed genes in biological processes were mainly enriched in transcription regulation, positive transcription regulation by RNA polymerase II, and signal transduction. Genes such as *SPI1*, *HOXD10*, and *UNC5C* were highly expressed during SE events, and genes such as *LHX2*, *CCL5*, and *HMX3* were highly expressed during MXE events. The differential genes in the class of “cellular component” were mainly enriched in the cell membrane, nucleus, cytoplasm, and cell surface. Genes such as *TLE6*, *CALCA*, and *CASQ2* were highly expressed during SE events, and genes such as *PRLR*, *TMEFF2*, and *RABL6* were highly expressed during MXE events. In the “molecular function” category, genes were mainly enriched for protein binding, DNA binding, DNA-binding transcription factor activity, and RNA polymerase. Genes such as *MAP3K2*, *ANK1*, and *NWD2* were highly expressed during SE events, and genes such as *SOX2*, *ZBTB8B*, and *SNED1* were highly expressed during MXE events. The target gene *MEI1* was enriched in the meiotic *I* signaling pathway in a class of “biological process”.

#### 3.3.4. KEGG Pathway Enrichment Analysis of Differentially Expressed Genes

KEGG is a public database for genome decoding, the basic unit of which is a pathway [27,28,29]. To better understand the biological functions of the genes, enrichment of KEGG pathways for the differentially expressed genes was performed (Figure 7C). The differential genes were mainly enriched in cytokine-cytokine receptor interaction, calcium signaling pathway, and fatty acid biosynthesis, which are related to spermatogenesis and cell proliferation. Genes such as *IL31RA*, *ATP2B3*, and *CASQ2* were highly expressed during SE events, whereas *IL11*, *PRLR*, and *CCR7* were highly expressed during MXE events. The distribution of up- and downregulation of differentially expressed genes across pathways was then further analyzed (Figure 7D). Cytokine-cytokine receptor interactions and fatty acid biosynthesis were mainly dominated by genes with downregulated expression.

### 3.4. Divergent Impacts of MEI1 SE and MXE Isoforms on the Cellular Metabolome Revealed by Comparative Metabolomics

#### 3.4.1. PCA and PLS-DA of Metabolites

**Figure 8:**
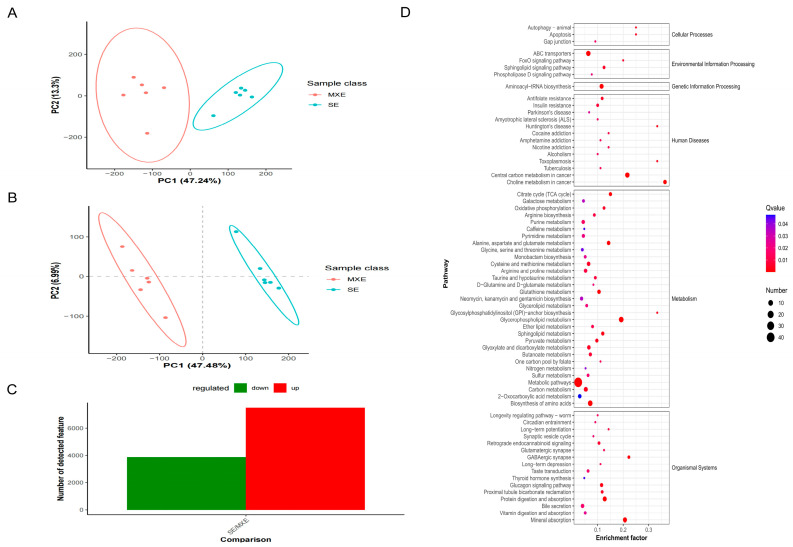
Screening, PCA, and KEGG enrichment analysis of differential metabolites. (**A**,**B**) Principal component analysis (PCA) score plots.The scatter plots (**A**,**B**) show the distribution of samples along the first (PC1) and second (PC2) principal components, colored by experimental groups. The ellipses in (**B**) represent the 95% confidence ellipse for each group (not shown for groups with biological replicates *n* < 4). (**C**) Bar chart displaying the number of differential metabolites. (**D**) The Rich Factor, calculated as the number of differential metabolites in a given KEGG pathway divided by the total number of metabolites assigned to that pathway, is represented on the x-axis. Each point’s color intensity corresponds to the enrichment *p*-value, with more significant (smaller) *p*-values indicating a higher degree of KEGG pathway enrichment.

The identified metabolites were analyzed using PCA and PLS-DA. Each point in the figure represents a sample, with the clustering of points indicating a high degree of similarity in the observed variables and the discrete points representing significant differences in the observed variables. PCA showed a clear separation between the MEI1 (MXE) and MEI1 (SE) groups, indicating that there are differences between the metabolites of the two groups; therefore, they can be used for further analysis (Figure 8A). PLS-DA showed that the MEI1 (MXE) and MEI1 (SE) groups were effectively separated. They were located on both sides of the centroid and exhibited a clear differentiation effect (Figure 8B).

#### 3.4.2. Results of Differential Metabolites Screening

For the statistical tests, univariate analysis of the fold-change and the *t*-test were used, and the q-value was determined using BH correction. Combined with the variable importance for projection (VIP) value obtained by PLS-DA, multivariate statistical analysis was used to search for differentially expressed metabolites. The following conditions were met: ratio ≥ 1.5, or ratio ≤ 1/1.5; *p*-value ≤ 0.05; and VIP ≥ 1. Using MEI1 (MXE) as the control group, 11360 differential metabolites were screened in the MEI1 (SE) group, of which 7494 had upregulated and 3866 had downregulated expression (Figure 8C and Figure S5).

#### 3.4.3. KEGG Pathway Enrichment Analysis of Differential Metabolites

To better understand the biological functions of the differential metabolites, KEGG pathway enrichment analysis was performed on the selected differential metabolites (Figure 8D). The differential metabolites were mainly enriched in metabolic pathways related to spermatogenesis, such as the citrate cycle, folate biosynthesis, and thyroid hormone synthesis. Among the above metabolic pathways, folate and spermine were highly expressed during SE events, and citrate and glutathione were highly expressed during MXE events.

### 3.5. Integrative Analysis of Transcriptomic and Metabolomic Data Reveals Molecular Networks Underlying MEI1 Isoform-Specific Functions

#### 3.5.1. Correlation and Enrichment Analysis of Differential Genes and Differential Metabolites

**Figure 9:**
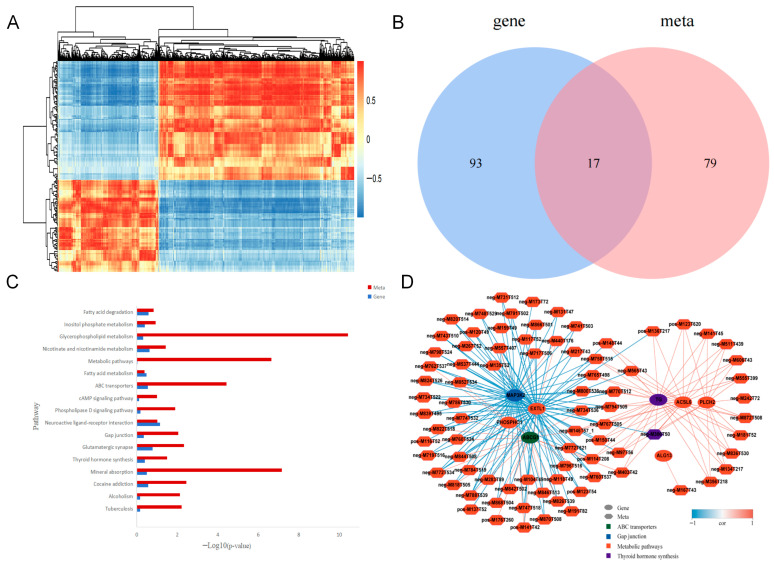
Joint analysis of differential genes and differential metabolites. (**A**) Clustered heatmap of correlation coefficients between differentially expressed genes (DEGs) and differential metabolites (DMs). The heatmap visualizes the Pearson correlation coefficients, demonstrating coordinated changes between the transcriptomic and metabolomic profiles. (**B**) Venn diagram identifying shared KEGG pathways. The diagram illustrates the intersection of KEGG pathways significantly enriched by DEGs and DMs, highlighting biological processes commonly perturbed at both molecular levels. (**C**) Bar plot of KEGG pathway enrichment. The bar chart displays the top significantly enriched KEGG pathways common to both DEGs and DMs. The x-axis represents the rich factor, while the y-axis lists the pathway names. (**D**) Correlation network of DEGs and DMs within four key pathways. The network depicts the interactions between DEGs (rectangular nodes) and DMs (circular nodes) within four selected pathways. Edges represent significant correlations, highlighting potential regulatory relationships. Solid blue lines indicate negative correlations and solid red lines indicate positive correlations.

Differential genes and metabolites were analyzed for correlation (Figure 9A). The correlation between differentially expressed genes and metabolites was high. Pathways that were enriched for both differential genes and metabolites were analyzed using a Venn diagram, which yielded an intersection, and differential genes and metabolites were found to be co-enriched for 17 pathways (Figure 9B). The KEGG pathway enrichment analysis revealed (Figure 9C) that the enriched pathways were metabolic pathways, fatty acid metabolism, ABC transporters, cAMP signaling, gap junctions, and thyroid hormone synthesis. As a source of energy for the sperm, fatty acids can ensure normal spermatogenesis. The mammalian sperm membrane is rich in polymeric fatty acids, and fatty acid metabolism is closely related to spermatogenesis [30,31,32]. The ABC transporter protein is an important component of the cell, playing the role of a “gatekeeper” and ensuring that beneficial substances enter the cell and harmful substances are prevented from entering the cell. The cAMP signaling pathway is the second intracellular signal found, with the cAMP-protein kinase A signaling pathway regulating sperm energy metabolism and playing an important role in spermatogenesis [33]. The gap junction is located in the cell membrane in connecting pathways between neighboring cells and plays an important role in the initiation of spermatogenesis. Thyroid hormones play an important role in the testes, and changes in the thyroid status are closely related to spermatogenesis [34]. Of the differential genes and metabolites involved in the co-enrichment pathway, *ALG13*, *CALCA*, *MAP3K2*, and *ATP2B3* were highly expressed during SE events, whereas *ACSL6*, *PLCH2*, *TG*, and *ABCG1* were highly expressed during MXE events. Folic acid, spermine, and phosphocreatine were strongly expressed during SE events, whereas glutamic acid, glutathione, and citric acid were strongly expressed during MXE events.

#### 3.5.2. Correlation Network Diagram Analysis of Differential Genes and Differential Metabolites

The relationships between the metabolites and genes were illustrated using network diagrams. Correlation network diagrams of differential genes and differential metabolites were analyzed for the four pathways in co-enrichment (Figure 9D). The gene *EXTL1* with upregulated expression in the metabolic pathway can positively regulate the production of folic acid, which can affect the expression of spindle checkpoints and the production of spermatogonocytes. The gene *ACSL6* with downregulated expression positively regulates the production of glutathione, an antioxidant that reduces the effects of oxidative stress on sperm and thus improves sperm quality.

## 4. Discussion

This study focuses on investigating *MEI1* alternative splicing in testicular Sertoli cells based on two primary considerations. First, Sertoli cells play a pivotal role in regulating spermatogenesis: beyond forming the blood-testis barrier, they secrete various cytokines, androgen-binding proteins, and essential metabolites (such as folate and fatty acids) that provide nutritional support for spermatogonia and spermatocytes, thereby critically governing the initiation and progression of spermatogenesis. Second, Sertoli cells offer unique experimental advantages: unlike germ cells, primary Sertoli cells can be efficiently cultured and genetically manipulated in vitro, enabling precise dissection of the transcriptomic and metabolomic characteristics of the SE and MXE *MEI1* splice variants within a pure cellular context.

*MEI1* was the first meiosis-specific mutant gene identified by forward genetics in mammals. Alternative splicing is a common biological process in eukaryotes and is of great significance for studying biological phenomena such as growth, reproduction, and signal transduction in eukaryotes [35]. Our previous study found that there are two alternative splicing modes of MXE and SE in the *MEI1* gene in the testicular tissue of Mongolian horses after sexual maturity, suggesting that the alternative splicing pattern of the *MEI1* gene might be related to spermatogenesis [16]. Therefore, in this study, using *MEI1* as the target gene, the effects of the alternative splicing events MXE and SE in the *MEI1* gene on spermatogenesis in Mongolian horses were investigated through a series of experiments, where MEI1 (MXE) was the control group and MEI1 (SE) was the experimental group.

As a new deep sequencing technology, RNA-Seq technology is widely used in genome research; for example, for differential gene screening and functional annotation, the complex functional properties of the transcriptome can be analyzed. Transcriptomic analysis of the reproductive organs of pigs has demonstrated the high efficiency of RNA-Seq [3,36]. Metabolomics is the scientific study of small chemical molecules with a molecular weight of less than 1000 Da that occur in cells, organs, or organisms and are involved in metabolism. Metabolomics is a new technique for identifying changes in endogenous metabolites in response to internal and external factors (such as the environment and disease). They are divided into targeted and untargeted metabolomics [37]. Metabolomics has the advantage that there are fewer types of metabolites than other histologies; metabolite analysis provides insight into the pathological state of the organism, reflects and amplifies small changes in gene and protein expression, and uses more versatile techniques [38]. Multi-omics techniques have also been widely used. Zhao [39] identified differential genes and metabolites after tretinoin exposure by combined transcriptome and metabolome analyses and further investigated the possible metabolic changes and molecular mechanisms. Liu [40] analyzed the condition of rice before and after infestation by a stem borer, and the results provided a new direction for the study of the defense mechanism of rice. In this study, RNA-Seq and metabolomics technologies were used to study Mongolian horse testis Sertoli cells with MXE and SE events of the *MEI1* gene. Both differentially expressed genes and metabolites were employed in the joint analysis. The mechanism of *MEI1* gene regulation by different alternative splicing events in Mongolian horse spermatogenesis was investigated at the transcriptome and metabolome levels.

A total of 193 differentially expressed genes were screened by RNA-Seq, comprising 109 genes such as *MEI1* and 84 genes, with upregulated and downregulated expression, respectively, suggesting that *MEI1* undergoes SE events to promote its own expression. The differentiated genes were mainly enriched in pathways related to spermatogenesis, such as regulating the proliferation and development of epithelial cells and their division and regulating transcription and protein binding. The spermatogenic epithelium contains spermatogenic cells and Sertoli cells and consists of basal and proximal chambers. Spermatogenesis is a collective term for various germ cells whose main function is to continuously produce sperms. Sertoli cells support, protect, and nourish germ cells [41]. The ABPs secreted by Sertoli cells bind testosterone to ensure spermatogenesis. The target gene, *MEI1*, was enriched in GO in the meiotic I pathway of biological processes. Other genes, such as *SPI1*, *TLE6*, and *MAP3K2*, were highly expressed in SE events, while *LHX2*, *PRLR*, and *SOX2* were highly expressed in MXE events. KEGG pathway enrichment analysis revealed that the differentially expressed genes were enriched in pathways related to spermatogenesis and cell proliferation, such as cytokine-cytokine receptor interactions, the calcium signaling pathway, and fatty acid biosynthesis. The binding of cytokines to their receptors triggers complex cytokine-cytokine receptor interactions that ultimately lead to changes in gene transcription, and cytokines regulate cell growth and differentiation during the immune response. The calcium signaling pathway promotes cell proliferation and triggers apoptosis. The calcium signaling function of *ANXA7* plays an important role in preventing tumorigenesis [42]. Fatty acids are involved in energy metabolism and sperm motility. Fetal BPA exposure inhibits endogenous long-chain fatty acid metabolism in adult male testes, which ultimately impairs sperm quality [43]. By determining the differentially expressed genes of the MEI1(MXE) and MEI1(SE) groups and analyzing them by GO and KEGG enrichment analyses, it was found that both alternative splicing events could promote cell proliferation and spermatogenesis, with the SE event promoting the expression of the target gene *MEI1*.

Metabolomic data were analyzed to screen for 11,360 distinct metabolites, of which the expression levels of 7494 were upregulated, and those of 3866 were downregulated. KEGG enrichment analysis revealed that the differential metabolites were mainly enriched in several metabolic pathways related to spermatogenesis, such as metabolic pathways, the citric acid cycle, folic acid biosynthesis, and thyroid hormone synthesis. The citrate cycle serves as a central metabolic pathway and is closely associated with spermatogenesis. Studies have demonstrated that specific knockout of key transporters in this pathway leads to significantly reduced metabolite levels within the cycle [44]. Folic acid is a B vitamin that is mainly absorbed in the small intestine and can influence the number and activity of sperms. Supplementation of folic acid is beneficial for maintaining the endocrine function of the ovaries. Thyroid hormone (TH) acts synergistically with FSH to induce and activate the synthesis of aromatase, a key enzyme for estrogen production in the ovaries, which converts androgens to estrogen. Among the metabolites associated with spermatogenesis, folic acid and spermine were highly expressed during SE events, and citric acid and glutathione were highly expressed during MXE events. By detecting the different metabolites in the MEI1(MXE) and MEI1(SE) groups, it was found that both SE and MXE events in the *MEI1* gene contribute to spermatogenesis to some extent.

Finally, joint analysis of the transcriptome and metabolome revealed a high correlation of differential genes and metabolites as well as co-enrichment with 17 signaling pathways, such as the metabolic pathway, fatty acid metabolism, cAMP signaling pathway, and TH synthesis, all of which are closely linked to the process of spermatogenesis. Fatty acids are divided into saturated fatty acids and unsaturated fatty acids, and unsaturated fatty acids are divided into monounsaturated fatty acids and polyunsaturated fatty acids. Oleic acid, an unsaturated fatty acid, combines with triglycerides and phospholipids to provide energy for the acrosomal reaction [45]. There was a small difference in fatty acid concentrations between the testes and sperm cells. The fatty acid composition in the testes determines spermatogenesis as well as the degree of acrosome reaction and sperm-egg binding, which are closely related to male reproduction. If fatty acid composition is altered, sperm viability is impaired, leading to infertility [46,47]. TH is synthesized and secreted by follicular epithelial cells to maintain mitochondrial function, oxidative stress, and DNA integrity. Abnormal TH levels (either high or low) can affect male fertility. High levels are associated with decreased sperm density and motility; low levels decrease forward motility and affect sperm morphology but do not affect other hormone levels or sperm density. The increase in TH levels has a more pronounced effect on sperm [48]. Integrated multi-omics analysis reveals that distinct splicing isoforms of *MEI1* indirectly regulate spermatogenesis by systematically rewiring signaling and metabolic networks in Sertoli cells. The cAMP signaling pathway emerges as a critical hub in this process. Specifically, in the skipped exon (SE) event, we observed significant upregulation of *MAP3K2* expression. Previous studies have established that altered *MAP3K2* expression is closely associated with modulation of the G protein/cAMP/PKA signaling pathway [49]. Research by Zhou et al. [50]. in oocytes demonstrated that cAMP/PKA signaling inhibits meiotic progression by affecting Mos protein accumulation, potentially through cAMP-mediated suppression of MAPK phosphorylation. Building on this evidence, we hypothesize that in Mongolian horse Sertoli cells, the potentially enhanced cAMP/PKA signaling driven by the *MEI1* SE isoform may similarly stabilize Mos or other intermediary molecules, thereby promoting *MAP3K2* activation and subsequent MAPK signaling cascades. Furthermore, *ACSL6* was found to be highly expressed in the mutually exclusive exon (MXE) event and significantly enriched in fatty acid metabolic pathways. *ACSL6* encodes long-chain acyl-CoA synthetase, which catalyzes the conjugation of long-chain fatty acids with coenzyme A to form acyl-CoA—a crucial initial step directing fatty acids toward β-oxidation or incorporation into triglycerides and phospholipids [51]. This process bears dual significance for spermatogenesis: first, in energy supply—activated acyl-CoA enters mitochondria for β-oxidation, generating substantial ATP to fuel energy-intensive activities of germ cells, particularly sperm; second, in structural assembly—fatty acids serve as essential components of phospholipids, which constitute the core structural basis of sperm membrane systems, including the acrosomal and mitochondrial membranes. Notably, dysregulation of fatty acid synthesis and activation pathways has been observed in Sertoli cells of patients with non-obstructive azoospermia, accompanied by significantly reduced *ACSL1* expression, underscoring the importance of lipid metabolic homeostasis in male reproduction [52]. Additional potential regulatory mechanisms were identified in this study. For instance, upregulation of *EXTL1* may positively regulate folate synthesis, influencing spindle checkpoint function and ultimately modulating spermatogenesis. Conversely, downregulated *ACSL6* expression may enhance glutathione biosynthesis, strengthening antioxidant defense capacity, mitigating oxidative damage to sperm, and thereby improving sperm quality.

The question of whether the MXE/SE splicing patterns of the *MEI1* gene observed in Mongolian horse Sertoli cells exhibit species specificity remains to be fully explored. Although the basic mechanisms of MXE and SE splicing are evolutionarily conserved across mammals, the functional consequences of these events are likely determined by the specific exons involved and the resulting protein isoforms. This study demonstrates that these distinct *MEI1* splicing variants are linked to different downstream pathways—the SE variant is associated with the cAMP signaling pathway, while the MXE variant is connected to fatty acid metabolism through *ACSL6*. This suggests that the regulatory logic of *MEI1* splicing may have undergone adaptive evolution to fine-tune spermatogenesis across different species. We hypothesize that although the core meiotic function of this gene is universal, its splicing regulation in somatic components of the testis (such as Sertoli cells) may have evolved to meet the unique physiological demands of different mammalian lineages. For instance, the specific exons skipped in SE events, or the exon pairs involved in MXE events, may differ between species, leading to isoforms with distinct functional properties. In summary, while a definitive answer regarding species specificity requires further comparative studies, our findings provide a foundation for future investigations into the evolutionary adaptation of *MEI1* splicing in mammalian reproduction.

However, our reliance on a primary Sertoli-cell in vitro model entails certain limitations: First, the monoculture lacks the complex interplay with germ cells and other testicular cell types, making it difficult to fully recapitulate the in vivo spermatogenic microenvironment. Second, although we have quantified transcriptomic and metabolomic changes, these have not yet been directly correlated with spermatogenic phenotypes (e.g., sperm count, motility, and morphology); to address this, we plan to employ a Sertoli–spermatogonial stem cell co-culture system to assess the effects of *MEI1* SE/MXE isoforms on meiotic progression and sperm function. Third, comprehensive validation in intact testicular tissue is warranted: we will apply RNA in situ hybridization or spatial transcriptomics to map the spatial distribution of each isoform across Sertoli cells, spermatogonia, and spermatocytes, and combine this with Sertoli-cell-specific transgenic animal models to further elucidate their impact on male fertility.

## 5. Conclusions

In this study, we investigated the molecular effects of two alternative splice variants of the *MEI1* gene (MXE and SE) in cultured Mongolian horse Sertoli cells. Transcriptomic and metabolomic analyses revealed distinct and shared pathways potentially related to spermatogenesis. While these findings provide new insights into how *MEI1* alternative splicing may influence the Sertoli cell microenvironment, direct evidence of its role in sperm production remains to be established. This work lays a foundation for future in vivo studies to validate the physiological relevance and explore potential applications in equine reproductive improvement.

## Data Availability

The accession number for the raw and processed profiling by high-throughput RNA sequencing technology reported in this paper is GEO: GSE254391.

## Supplementary Materials

The following supporting information can be downloaded at: https://www.mdpi.com/article/10.3390/ani15233435/s1, Figure S1: Map of positive (left) negative (right) ion metabolism; Figure S2: Correlation analysis nine quadrants; Figure S3: Plasmid profile of MEI1 (MXE) in pGWLV12 (mcherry); Figure S4: Plasmid profile of MEI1 (SE) in pGWLV10-BFP; Figure S5: Heatmap showing the abundance profiles of differential metabolites; Table S1: Reverse transcription system; Table S2: Response Procedures; Table S3: Primer Sequence Information; Table S4: qRT-PCR Amplification System; Table S5: Response Procedures; Table S6: Sample test results; Table S7: Statistics of sequencing output; Table S8: Statistics of comparison efficiency.
