# Supplementary material for: The Impact of MEI1 Alternative Splicing Events on Spermatogenesis in Mongolian Horses
Source: Animals (Basel). 2025 Nov 28;15(23):3435. doi: 10.3390/ani15233435 (PMC12691261; doi:10.3390/ani15233435)
Supplement: Supplementary file 1 [file animals-15-03435-s001.zip › animals-3958610-supplementary/Supplementary Figure S1.pdf]

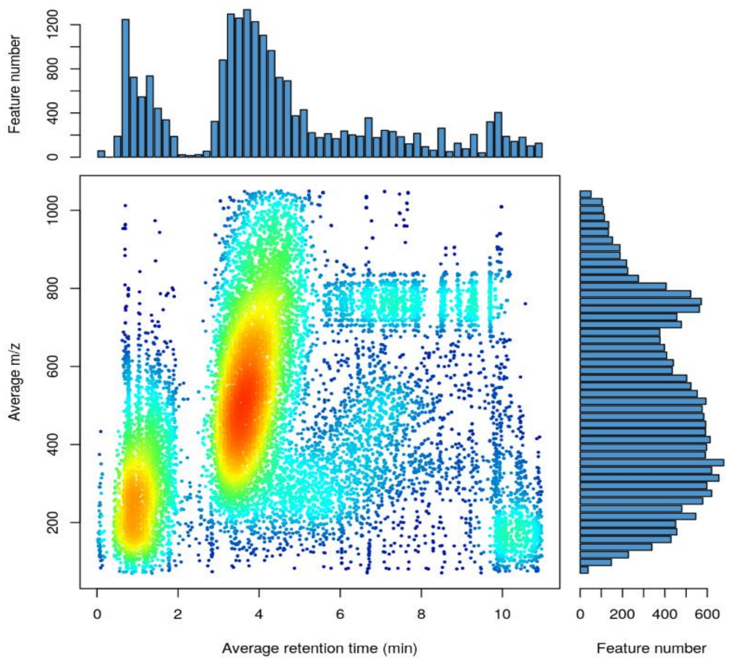

Fig.S1 Map of positive (left) negative (right) ion metabolism.Each point in the graph represents a substance, and the color indicates how dense the substance is in that area; The darker the color, the larger the number of Feature numbers.
