# Supplementary material for: The Impact of MEI1 Alternative Splicing Events on Spermatogenesis in Mongolian Horses
Source: Animals (Basel). 2025 Nov 28;15(23):3435. doi: 10.3390/ani15233435 (PMC12691261; doi:10.3390/ani15233435)
Supplement: Supplementary file 1 [file animals-15-03435-s001.zip › animals-3958610-supplementary/Supplementary Figure S2.pdf]

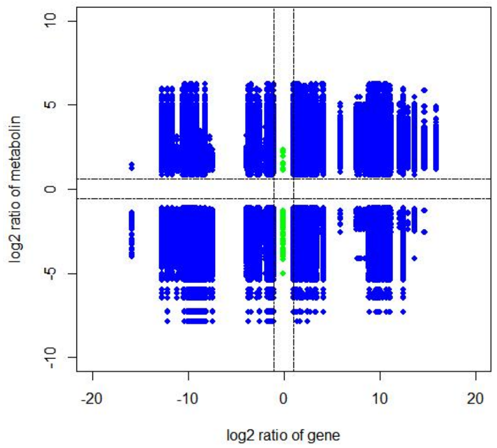

Fig.S2 Correlation analysis nine quadrants.The plot is divided by black dashed lines into nine quadrants (numbered 1 - 9 from left to right and top to bottom), representing different expression patterns of genes and metabolites: Quadrants 1 & 2, upregulated metabolites with downregulated or unchanged genes; Quadrant 4, downregulated genes with unchanged metabolites; Quadrant 6, upregulated genes with unchanged metabolites; Quadrants 8 & 9, downregulated metabolites with unchanged or upregulated genes; Quadrant 5, both unchanged; Quadrants 3 & 7, co-upregulated and co-downregulated pairs, respectively.
