# Supplementary material for: The Impact of MEI1 Alternative Splicing Events on Spermatogenesis in Mongolian Horses
Source: Animals (Basel). 2025 Nov 28;15(23):3435. doi: 10.3390/ani15233435 (PMC12691261; doi:10.3390/ani15233435)
Supplement: Supplementary file 1 [file animals-15-03435-s001.zip › animals-3958610-supplementary/Supplementary Materials Table 1.pdf]

Table.S1 Reverse transcription system

Reagent Name	Concentration
5×PrimeScript RT Master Mix(Perfect Real Time)	2 μL
Total RNA	1 μL
RNase Free dH2O	7 μL
Total	10 μL
