# Supplementary material for: The Impact of MEI1 Alternative Splicing Events on Spermatogenesis in Mongolian Horses
Source: Animals (Basel). 2025 Nov 28;15(23):3435. doi: 10.3390/ani15233435 (PMC12691261; doi:10.3390/ani15233435)
Supplement: Supplementary file 1 [file animals-15-03435-s001.zip › animals-3958610-supplementary/Supplementary Materials Table 2.pdf]

Table.S2 Response Procedures

Ncubation time	Incubation temperature
37 ℃	15 min（Reverse transcription）
85 ℃	5 sec（Reverse transcriptase inactivation）
4 ℃	∞
