# Supplementary material for: The Impact of MEI1 Alternative Splicing Events on Spermatogenesis in Mongolian Horses
Source: Animals (Basel). 2025 Nov 28;15(23):3435. doi: 10.3390/ani15233435 (PMC12691261; doi:10.3390/ani15233435)
Supplement: Supplementary file 1 [file animals-15-03435-s001.zip › animals-3958610-supplementary/Supplementary Materials Table 3.pdf]

Table.S3 Primer Sequence Information

Gene	Name of prime	Primer sequence（5′-3′）	Product length/bp
MEI1	Forward	F: GATGCTAACCGTGCTCTCCAAGTG	24
	Reverse	R: AGTCCTCCAGGGTGTCTTCTTTCTC	25
GAPDH	Forward	F: TCGGAGTGAACGGATTTG	18
	Reverse	R:CCTGGAAGATGGTGATGG	18
