# Supplementary material for: The Impact of MEI1 Alternative Splicing Events on Spermatogenesis in Mongolian Horses
Source: Animals (Basel). 2025 Nov 28;15(23):3435. doi: 10.3390/ani15233435 (PMC12691261; doi:10.3390/ani15233435)
Supplement: Supplementary file 1 [file animals-15-03435-s001.zip › animals-3958610-supplementary/Supplementary Materials Table 4.pdf]

Table.S4 qRT-PCR Amplification System

Reagent Name	Concentration
TB Green Premix Ex Taq II	12.5 μL
PCR Forward Primer	1.0 μL
PCR Reverse Primer	1.0 μL
cDNA	2.0 μL
ddH_2O	8.5 μL
Total	25 μL
