# Supplementary material for: The Impact of MEI1 Alternative Splicing Events on Spermatogenesis in Mongolian Horses
Source: Animals (Basel). 2025 Nov 28;15(23):3435. doi: 10.3390/ani15233435 (PMC12691261; doi:10.3390/ani15233435)
Supplement: Supplementary file 1 [file animals-15-03435-s001.zip › animals-3958610-supplementary/Supplementary Materials Table 5.pdf]

Table.S5 Response Procedures

	ncubation time	Incubation temperature
Pre-incubation Step	95 ℃	30 sec
Amplification Step	95 ℃	5 sec
（40 cycle）	60 ℃	30 sec
	95 ℃	15 sec
Annealing Temperature	60 ℃	30 sec
	95 ℃	15 sec
Hold Temperature	4 ℃	∞
