# Supplementary material for: The Impact of MEI1 Alternative Splicing Events on Spermatogenesis in Mongolian Horses
Source: Animals (Basel). 2025 Nov 28;15(23):3435. doi: 10.3390/ani15233435 (PMC12691261; doi:10.3390/ani15233435)
Supplement: Supplementary file 1 [file animals-15-03435-s001.zip › animals-3958610-supplementary/Supplementary Materials Table 6.pdf]

Table.S6 Sample test results

eceiving Date	Customer Supplied Information				LC Measurement						Original ID	LC Internal Record		
	Internal Sample ID	Sample Type	Species	Sample Detail Description	O.D. 260/280	O.D.260/230	nanodrop (μg/μL)	Amount (μg)	QC Evaluation	Reasons for disqualification		Isolation Method	Operator	Extraction Date
2023-04-24 14:10:52	23D25R-126 SE_1	Routinely cultured cells	马		2.10	1.70	0.61	25.67	Qualified		MEI1(SE)	TRK1002U	yuhp	2023-04-24 18:45:37
2023-04-24 14:10:52	23D25R-127 SE_2	Routinely cultured cells	马		2.16	1.71	0.19	8.05	Qualified		MEI1(SE)	TRK1002U	yuhp	2023-04-24 18:45:37
2023-04-24 14:10:52	23D25R-128 SE_3	Routinely cultured cells	马		2.06	1.77	0.86	36.09	Qualified		MEI1(SE)	TRK1002U	yuhp	2023-04-24 18:45:37
2023-04-24 14:10:52	23D25R-129 MXE_1	Routinely cultured cells	马		2.10	1.97	0.43	18.21	Qualified		MEI1(MXE)	TRK1002U	yuhp	2023-04-24 18:45:37
2023-04-24 14:10:52	23D25R-130 MXE_2	Routinely cultured cells	马		2.07	1.79	0.53	22.12	Qualified		MEI1(MXE)	TRK1002U	yuhp	2023-04-24 18:45:37
2023-04-24 14:10:52	23D25R-131 MXE_3	Routinely cultured cells	马		2.09	2.01	0.56	23.40	Qualified		MEI1(MXE)	TRK1002U	yuhp	2023-04-24 18:45:37

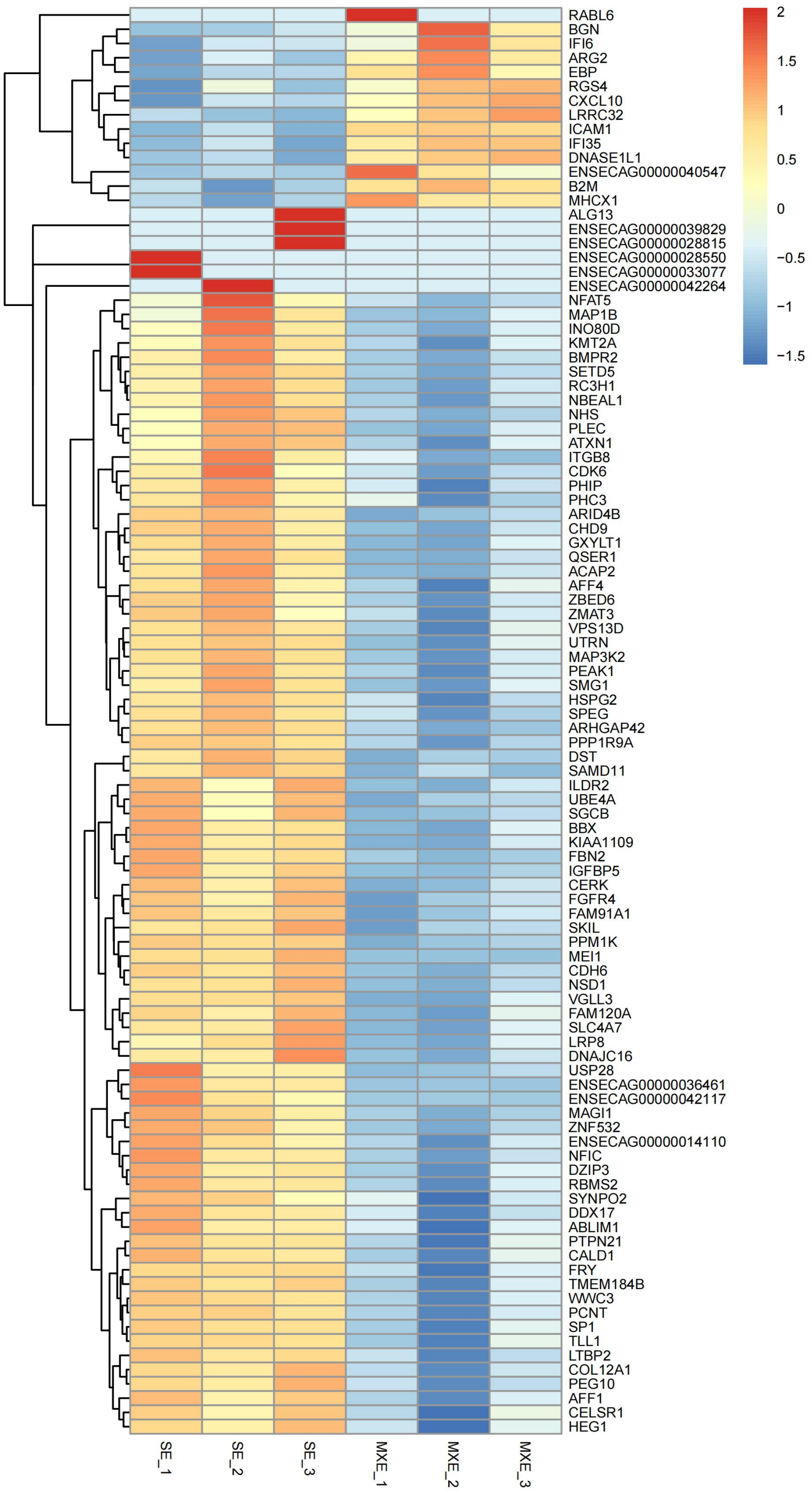
RABL6
BGN
IFI6
ARG2
EBP
RGS4
CXCL10
LRRC32
ICAM1
IFI35
DNASE1L1
ENSECAG00000040547
B2M
MHCX1
ALG13
ENSECAG00000039829
ENSECAG00000028815
ENSECAG00000028550
ENSECAG00000033077
ENSECAG00000042264
NFAT5
MAP1B
INO80D
KMT2A
BMPR2
SETD5
RC3H1
NBEAL1
NHS
PLEC
ATXN1
ITGB8
CDK6
PHIP
PHC3
ARID4B
CHD9
GXYLT1
QSER1
ACAP2
AFF4
ZBED6
ZMAT3
VPS13D
UTRN
MAP3K2
PEAK1
SMG1
HSPG2
SPEG
ARHGAP42
PPP1R9A
DST
SAMD11
ILDR2
UBE4A
SGCB
BBX
KIAA1109
FBN2
IGFBP5
CERK
FGFR4
FAM91A1
SKIL
PPM1K
MEI1
CDH6
NSD1
VGLL3
FAM120A
SLC4A7
LRP8
DNAJC16
USP28
ENSECAG00000036461
ENSECAG00000042117
MAGI1
ZNF532
ENSECAG00000014110
NFIC
DZIP3
RBMS2
SYNPO2
DDX17
ABLIM1
PTPN21
CALD1
FRY
TMEM184B
WWC3
PCNT
SP1
TLL1
LTBP2
COL12A1
PEG10
AFF1
CELSR1
HEG1
SE_1
SE_2
SE_3
MXE_1
MXE_2
MXE_3
2
1.5
1
0.5
0
−0.5
−1
−1.5

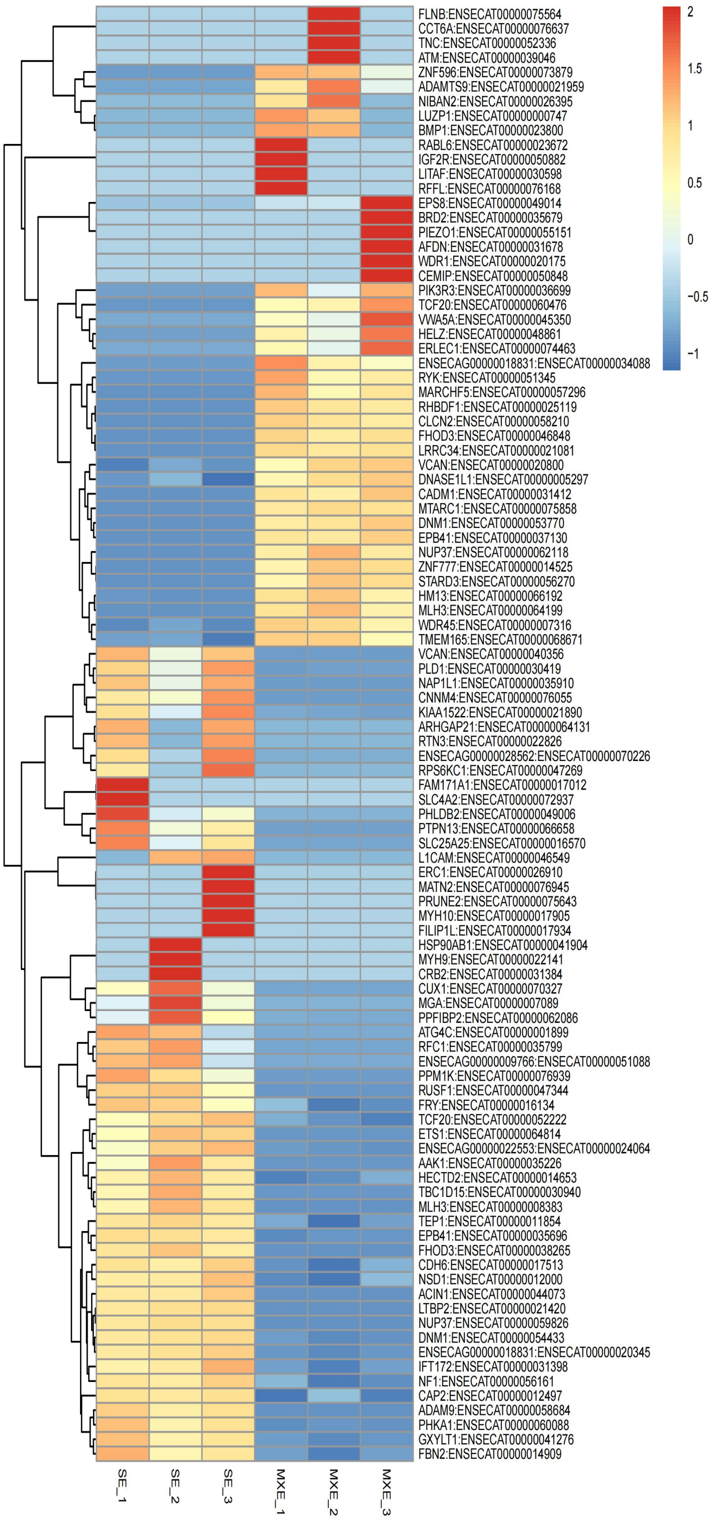
FLNB:ENSECAT00000075564
CCT6A:ENSECAT00000076637
TNC:ENSECAT00000052336
ATM:ENSECAT00000039046
ZNF596:ENSECAT00000073879
ADAMTS9:ENSECAT00000021959
NIBAN2:ENSECAT00000026395
LUZP1:ENSECAT00000000747
BMP1:ENSECAT00000023800
RABL6:ENSECAT00000023672
IGF2R:ENSECAT00000050882
LITAF:ENSECAT00000030598
RFFL:ENSECAT00000076168
EPS8:ENSECAT00000049014
BRD2:ENSECAT00000035679
PIEZO1:ENSECAT00000055151
AFDN:ENSECAT00000031678
WDR1:ENSECAT00000020175
CEMIP:ENSECAT00000050848
PIK3R3:ENSECAT00000036699
TCF20:ENSECAT00000060476
VWA5A:ENSECAT00000045350
HELZ:ENSECAT00000048861
ERLEC1:ENSECAT00000074463
ENSECAG00000018831:ENSECAT00000034088
RYK:ENSECAT00000051345
MARCHF5:ENSECAT00000057296
RHBDF1:ENSECAT00000025119
CLCN2:ENSECAT00000058210
FHOD3:ENSECAT00000046848
LRRC34:ENSECAT00000021081
VCAN:ENSECAT00000020800
DNASE1L1:ENSECAT00000005297
CADM1:ENSECAT00000031412
MTARC1:ENSECAT00000075858
DNM1:ENSECAT00000053770
EPB41:ENSECAT00000037130
NUP37:ENSECAT00000062118
ZNF777:ENSECAT00000014525
STARD3:ENSECAT00000056270
HM13:ENSECAT00000066192
MLH3:ENSECAT00000064199
WDR45:ENSECAT00000007316
TMEM165:ENSECAT00000068671
VCAN:ENSECAT00000040356
PLD1:ENSECAT00000030419
NAP1L1:ENSECAT00000035910
CNNM4:ENSECAT00000076055
KIAA1522:ENSECAT00000021890
ARHGAP21:ENSECAT00000064131
RTN3:ENSECAT00000022826
ENSECAG00000028562:ENSECAT00000070226
RPS6KC1:ENSECAT00000047269
FAM171A1:ENSECAT00000017012
SLC4A2:ENSECAT00000072937
PHLDB2:ENSECAT00000049006
PTPN13:ENSECAT00000066658
SLC25A25:ENSECAT00000016570
L1CAM:ENSECAT00000046549
ERC1:ENSECAT00000026910
MATN2:ENSECAT00000076945
PRUNE2:ENSECAT00000075643
MYH10:ENSECAT00000017905
FILIP1L:ENSECAT00000017934
HSP90AB1:ENSECAT00000041904
MYH9:ENSECAT00000022141
CRB2:ENSECAT00000031384
CUX1:ENSECAT00000070327
MGA:ENSECAT00000007089
PPFIBP2:ENSECAT00000062086
ATG4C:ENSECAT00000001899
RFC1:ENSECAT00000035799
ENSECAG00000009766:ENSECAT00000051088
PPM1K:ENSECAT00000076939
RUSF1:ENSECAT00000047344
FRY:ENSECAT00000016134
TCF20:ENSECAT00000052222
ETS1:ENSECAT00000064814
ENSECAG00000022553:ENSECAT00000024064
AAK1:ENSECAT00000035226
HECTD2:ENSECAT00000014653
TBC1D15:ENSECAT00000030940
MLH3:ENSECAT00000008383
TEP1:ENSECAT00000011854
EPB41:ENSECAT00000035696
FHOD3:ENSECAT00000038265
CDH6:ENSECAT00000017513
NSD1:ENSECAT00000012000
ACIN1:ENSECAT00000044073
LTBP2:ENSECAT00000021420
NUP37:ENSECAT00000059826
DNM1:ENSECAT00000054433
ENSECAG00000018831:ENSECAT00000020345
IFT172:ENSECAT00000031398
NF1:ENSECAT00000056161
CAP2:ENSECAT00000012497
ADAM9:ENSECAT00000058684
PHKA1:ENSECAT00000060088
GXYLT1:ENSECAT00000041276
FBN2:ENSECAT00000014909
SE_1
SE_2
SE_3
MXE_1
MXE_2
MXE_3
2
1.5
1
0.5
0
−0.5
−1
