# Supplementary material for: The Impact of MEI1 Alternative Splicing Events on Spermatogenesis in Mongolian Horses
Source: Animals (Basel). 2025 Nov 28;15(23):3435. doi: 10.3390/ani15233435 (PMC12691261; doi:10.3390/ani15233435)
Supplement: Supplementary file 1 [file animals-15-03435-s001.zip › animals-3958610-supplementary/Supplementary Materials Table 7.pdf]

Table.S7 Statistics of sequencing output

Sample	Raw Data		Valid Data		Valid Ratio(reads)	Q20%	Q30%	GC content %
	Read	Base	Read	Base				
MXE_1	40980004	6.15G	39669718	5.95G	96.80	99.97	97.40	50.0
MXE_2	44614212	6.69G	43323780	6.50G	97.11	99.97	97.58	49.0
MXE_3	41489028	6.22G	40088986	6.01G	96.63	99.97	97.37	49.5
SE_1	40619274	6.09G	39333844	5.90G	96.84	99.97	97.39	50.0
SE_2	41364862	6.20G	39948690	5.99G	96.58	99.97	97.42	50.0
SE_3	40866098	6.13G	39603146	5.94G	96.91	99.97	97.42	49.5
