# Supplementary material for: The Impact of MEI1 Alternative Splicing Events on Spermatogenesis in Mongolian Horses
Source: Animals (Basel). 2025 Nov 28;15(23):3435. doi: 10.3390/ani15233435 (PMC12691261; doi:10.3390/ani15233435)
Supplement: Supplementary file 1 [file animals-15-03435-s001.zip › animals-3958610-supplementary/Supplementary Materials Table 8.pdf]

Table.S8 Statistics of comparison efficiency

Sample	Valid reads	Mapped reads	Unique Mapped reads	Multiple Mapped reads
MXE-1	39669718	37092831(93.50%)	35998131(90.74%)	1094700(2.76%)
MXE-2	43323780	40773506(94.11%)	39528371(91.24%)	1245135(2.87%)
MXE-3	40088986	37415897(93.33%)	36308495(90.57%)	1107402(2.76%)
SE-1	39333844	36749603(93.43%)	35681714(90.72%)	1067889(2.71%)
SE-2	39948690	37366845(93.54%)	36278226(90.81%)	1088619(2.73%)
SE-3	39603146	37038271(93.52%)	35978443(90.85%)	1059828(2.68%)
