# Supplementary figures and images for: The Impact of MEI1 Alternative Splicing Events on Spermatogenesis in Mongolian Horses
Source: Animals (Basel). 2025 Nov 28;15(23):3435. doi: 10.3390/ani15233435 (PMC12691261; doi:10.3390/ani15233435)

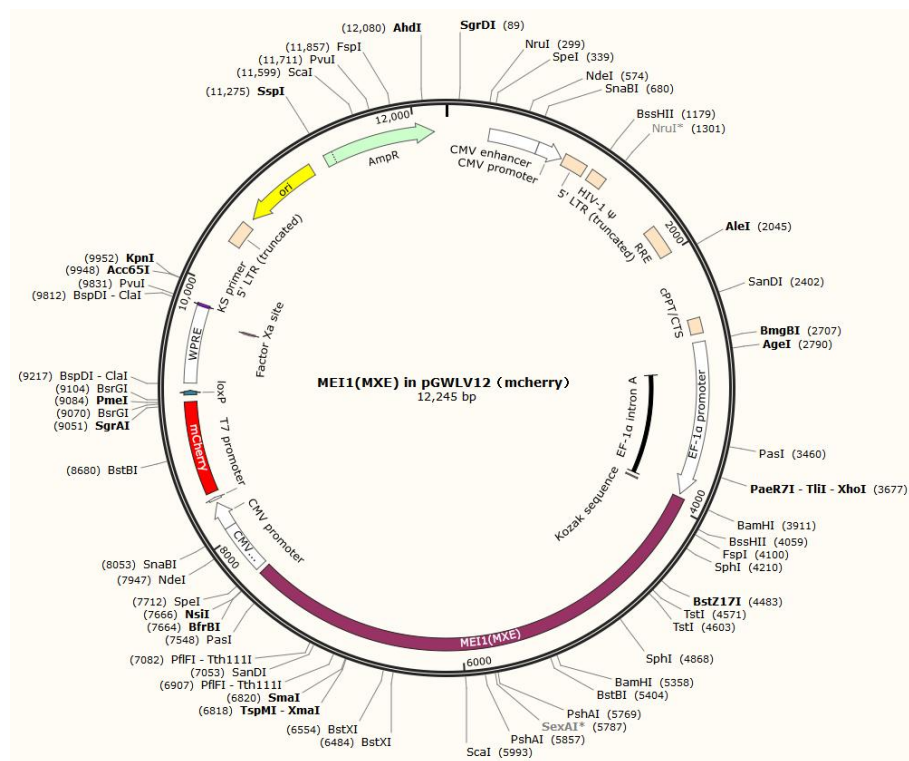

Fig. S3 Plasmid profile of MEI1 (MXE) in pGWL12 (mcherry) .

Supplement: Supplementary file 1 [file animals-15-03435-s001.zip › animals-3958610-supplementary/Supplementary Figure S3.pdf]

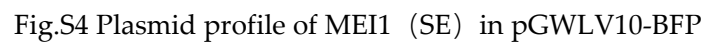

Fig.S4 Plasmid profile of MEI1 (SE) in pGWL V10-BFP

Supplement: Supplementary file 1 [file animals-15-03435-s001.zip › animals-3958610-supplementary/Supplementary Figure S4.pdf]

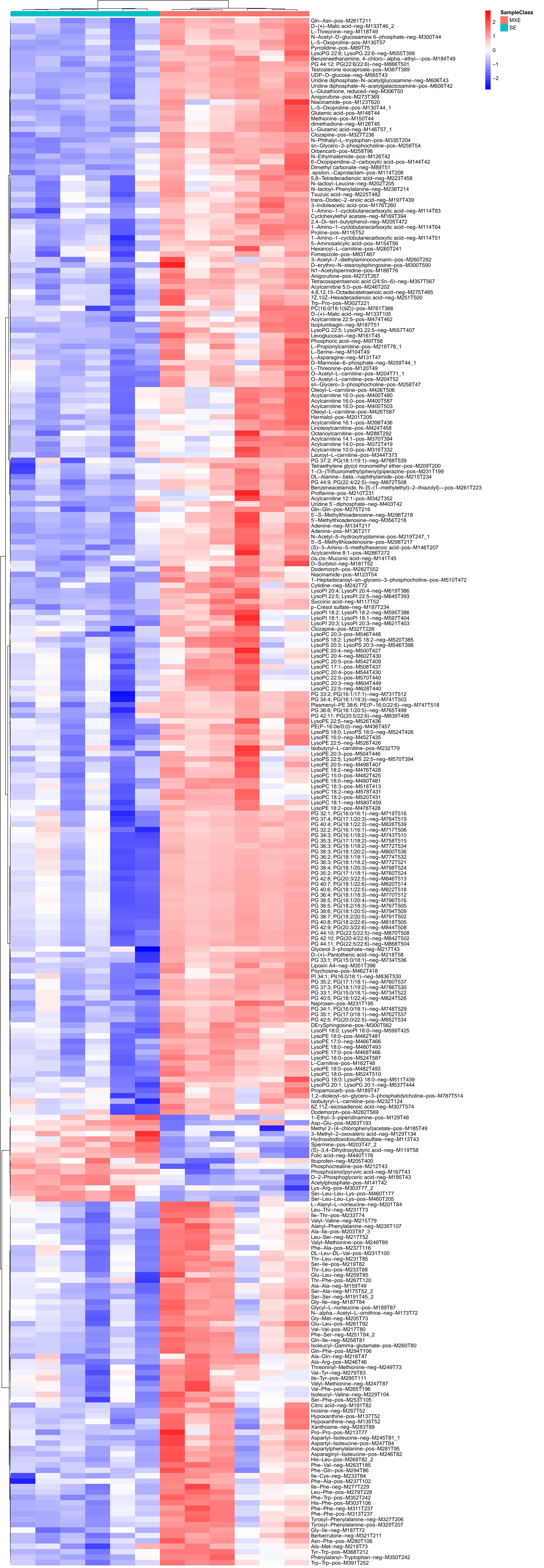

Supplement: Supplementary file 1 [file animals-15-03435-s001.zip › animals-3958610-supplementary/Supplementary Figure S5.pdf]
